# MicroRNA profiling and their pathways in South African individuals with prediabetes and newly diagnosed type 2 diabetes mellitus

**DOI:** 10.18632/oncotarget.25271

**Published:** 2018-07-17

**Authors:** Tandi E. Matsha, Andre P. Kengne, Stanton Hector, Desiree L. Mbu, Yandiswa Y. Yako, Rajiv T. Erasmus

**Affiliations:** ^1^ Department of Biomedical Sciences, Faculty of Health and Wellness Science, Cape Peninsula University of Technology, Cape Town, South Africa; ^2^ Non-Communicable Diseases Research Unit, South African Medical Research Council, Cape Town, South Africa; ^3^ Department of Medicine, University of Cape Town, Cape Town, South Africa; ^4^ Department of Human Biology, Faculty of Health Sciences, Walter Sisulu University, Mthatha, South Africa; ^5^ Department of Pathology, Faculty of Health Sciences, National Health Laboratory Service (NHLS) and University of Stellenbosch, Cape Town, South Africa

**Keywords:** Africa, diabetes, miRNA, prediabetes, sequencing

## Abstract

Early identification of individuals with elevated risk of developing diabetes mellitus, followed by the implementation of effective prevention interventions can delay the onset of the disease and related complications. In this regard, recent studies have shown that miRNAs are useful as early markers of certain disease types, including diabetes. We used high throughput sequencing to assess miRNA expression profiles from whole blood of 12 individuals with screen-detected diabetes, 12 with prediabetes and 12 with normal glucose tolerance, matched for age, blood pressure, smoking and body mass index. We identified a total of 261 (57 novel) differentially expressed miRNA profiles between the study groups. Comparison of the miRNA expression profiles between prediabetess and diabetes revealed 25 common miRNA, but highlighted some interesting differences. For instance, three miRNAs (miR-126-3p, miR-28-3p miR-486-5p) were dysregulated in prediabetes compared to screen-detected diabetes. Target gene analysis showed thousands of potential genes and KEGG pathway analysis revealed 107 significant pathways of which some are involved signal transduction, cell-cell communications, cell growth and death, immune response, endocrine system and metabolic diseases. This first detailed African study has shown both known and novel differentially expressed miRNAs in relation to glucose tolerance.

## INTRODUCTION

Diabetes mellitus (DM) is a major cause of morbidity and mortality worldwide and has resulted in deaths of approximately five million people aged between 20 and 79 years in 2015 according to the International diabetes federation [1]. Sub-Saharan Africa (SSA) is currently experiencing the fastest relative growth of people with diabetes that remains undiagnosed in many [2]. In 2015, the International Diabetes Federation (IDF) Diabetes Atlas, estimated that nearly 2/3^rd^ of the 14.2 million sub-Saharan Africans adults with diabetes were undiagnosed [1]. Overt DM is often preceded by prediabetes, a term used to define subjects with either or both impaired fasting glucose (IFG) and impaired glucose tolerance (IGT). Approximately, 5–10% of individuals with prediabetes develop diabetes annually with up to 70% eventually progressing to the stage of the disease [3, 4]. Lifestyle modifications or drug interventions can delay the onset of DM [5, 6], however this is dependent on the identification of those at risk of progression to overt DM. This identification is heavily dependent on the diagnostic criteria which are continually revised and vary across stakeholders. For example, the World Health organisation (WHO) and American Diabetes Federation differ in the description of IFG, respectively 6.1 – 6.9 mmol/| and 5.6–6.9 mmol/L. Moreover in 2010 the ADA included HbA1c ranging from 5.7% to 6.4% as diagnostic criterion for prediabetes. These realities validate the critical need for earlier and more efficient biomarkers for diabetes risk screening. In this regard, recent studies [7–9] have shown that micro RNAs (miRNAs) are useful as early markers of certain disease types, including diabetes.

To date, over 1881 miRNA precursors that can generate over 2588 mature miRNAs have been identified in the human genome (www.mirbase.org) which control ∼50% of all mammalian protein-coding genes [10]. miRNAs are a class of small (∼19–24 nucleotides in length), endogenous, evolutionarily conserved RNAs that function as posttranscriptional regulators of gene expression [11–13]. They primarily function by binding to complementary target sequences in messenger RNA (mRNA) and by interfering with the translational machinery, thereby prevent or alter the production of the protein product [11–13]. In addition to repressing translation, miRNA binding to its target mRNA also triggers the recruitment and association of mRNA decay factors, leading to mRNA destabilization, degradation, and resultant decrease in expression levels [13]. Dysregulated expression of miRNAs is associated with the pathogenesis of a variety of diseases including dysglycaemia and vascular complications. Several miRNAs that are involved in the regulation of insulin signaling, lipid and glucose homeostasis have been identified in various tissues [14, 15], however, findings are inconsistent owing to techniques, sample and genetic diversity of populations studied [16, 17].

Despite the growing evidence of the important role and potential diagnostic value of miRNAs in dysglycaemia, such properties are yet to be demonstrated in the African setting. Therefore, in the present study we aimed to identify dysregulated miRNA in a South African mixed ancestry population previously reported to be at high risk of diabetes [18]. To avoid potential bias from treatment induced alterations in miRNA expression, we focused on individuals with normal glucose tolerance (NGT), prediabetes individuals with IGT only and those with screen-detected diabetes who had not initiated glucose lowering drug treatment.

## RESULTS

### General characteristics of participants

The characteristics of participants overall, and according to glucose tolerance status are summarised in Table 1. All participants were female and the mean levels of diastolic blood pressure, systolic blood pressure, low density lipoprotein cholesterol, high density lipoprotein cholesterol, age, body mass index, and serum cotinine did not differ significantly by status for glucose tolerance (all p≥0.06).

**Table 1:** Characteristics of the 36 women included overall, and by status for glucose tolerance

Characteristics	Total	Normal	IGT	New DM	p-value
n	36	12	12	12	
Age (years)	53.4 (7.8)	52.1 (7.8)	53.5 (8.5)	54.8 (7.5)	0.717
Weight (kg)	76.8 (19.7)	70.2 (18.9)	79.3 (21.7)	80.8 (18.3)	0.370
Height (cm)	155.8 (6.5)	157.2 (7.9)	154.4 (6.0)	156.1 (5.9)	0.600
Body mass index (kg/m^2^)	31.5 (8.4)	27.3 (5.8)	33.3 (9.1)	33.5 (8.9)	0.141
Waist circumference (cm)	93.9 (18.7)	83.2 (18.5)	97.1 (13.8)	101.3 (19.7)	0.041
Hip circumference (cm)	107.1 (17.7)	102.0 (18.4)	109.9 (18.6)	109.4 (16.6)	0.487
Waist/hip ratio	0.87 (0.08)	0.81 (0.07)	0.89 (0.06)	0.92 (0.07)	0.001
Systolic blood pressure (mmHg)	138.4 (27.5)	135.3 (30.8)	137.0 (18.5)	142.9 (32.9)	0.784
Diastolic blood pressure (mmHg)	87.0 (18.0)	78.0 (16.1)	88.9 (11.7)	94.2 (22.1)	0.077
Pulse rate (bpm)	72.1 (13.5)	64.8 (12.2)	71.3 (5.8)	80.1 (16.4)	0.016
Fasting plasma glucose (mmol/L)	6.3 (2.9)	4.6 (0.8)	5.3 (0.5)	9.1 (3.6)	< 0.0001
2-hour glucose (mmol/L)	10.5 (5.5)	5.3 (1.6)	9.3 (0.9)	16.5 (4.7)	< 0.0001
Fasting insulin (mIU/L)^*^	8.10 (5.70-12.50)	5.65 (2.95-7.85)	9.45 (7.55-12.50)	14.10 (5.90-21.20)	0.0058
2-hour Insulin (mIU/L)^*^	53.20 (29.20-97.90)	28.50 (17.80-40.40)	111.20 (60.20-187.20)	48.80 (31.45-83.30)	0.0044
Triglycerides (mmol/L)	1.6 (1.1)	1.0 (0.3)	1.76 (1.0)	2.2 (1.3)	0.023
LDL Cholesterol (Measured) (mmol/L)	3.6 (1.0)	3.3 (1.0)	3.5 (1.0)	4.2 (0.8)	0.060
HDL cholesterol (mmol/L)	1.4 (0.5)	1.6 (0.5)	1.3 (0.4)	1.4 (0.6)	0.451
Total cholesterol (mmol/L)	5.7 (1.0)	5.5 (1.1)	5.4 (1.0)	6.4 (0.8)	0.043
Total/HDL cholesterol ratio	4.3 (1.4)	3.7 (1.2)	4.3 (1.3)	5.02 (1.4)	0.077
High-sensitivity CRP (mg/L)	7.84 (8.56)	3.12 (2.73)	9.28 (5.93)	11.11 (12.34)	0.052
Cotinine (ng/mL)^*^	22.5 (10.0-220.5)	22.5 (10.0-261.0)	91.0 (10.0-220.0)	85.0 (10.0-206.0)	1.0000

^*^, median [25^th^-75^th^ percentiles), and p-values from Kruskal wallis test.

### miRNA expression profiling

**Figure 1:**
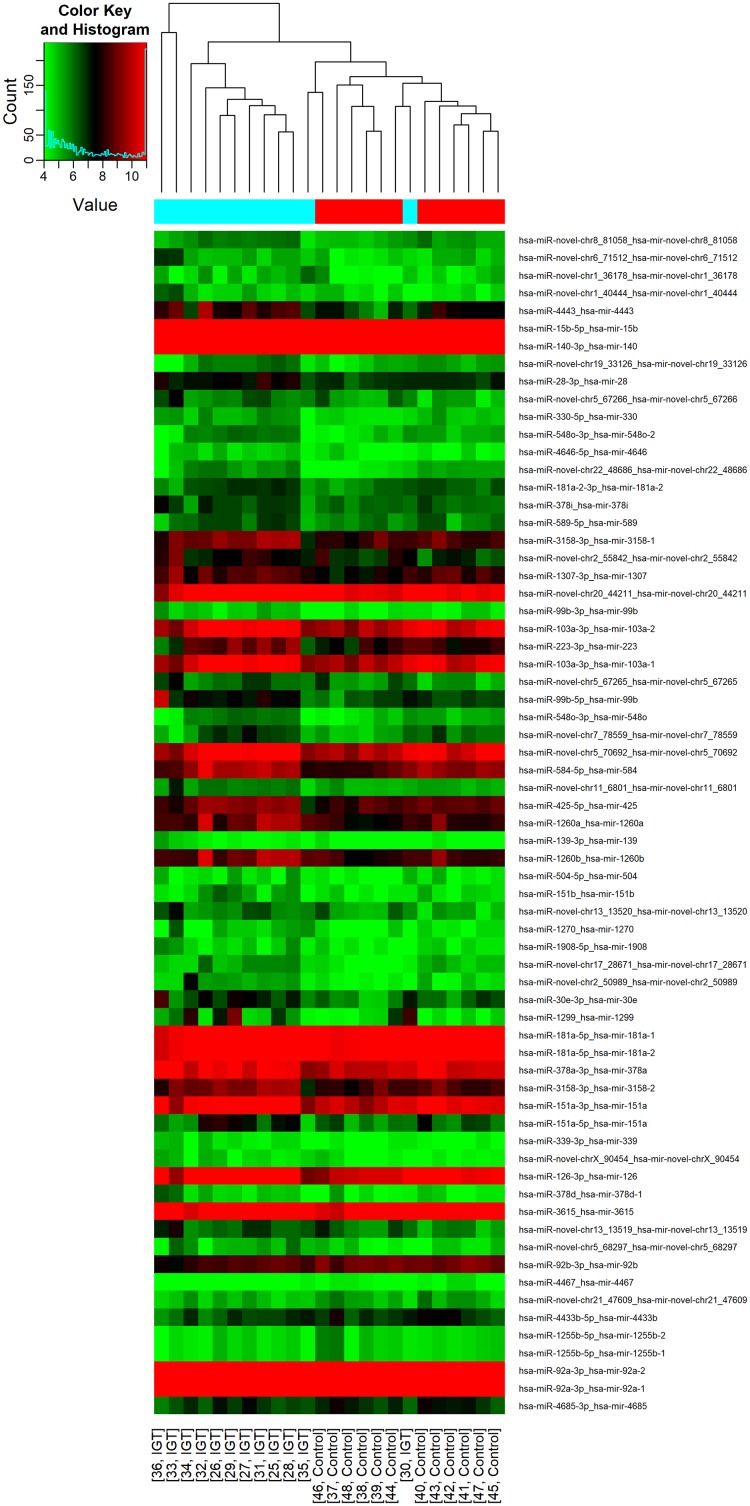

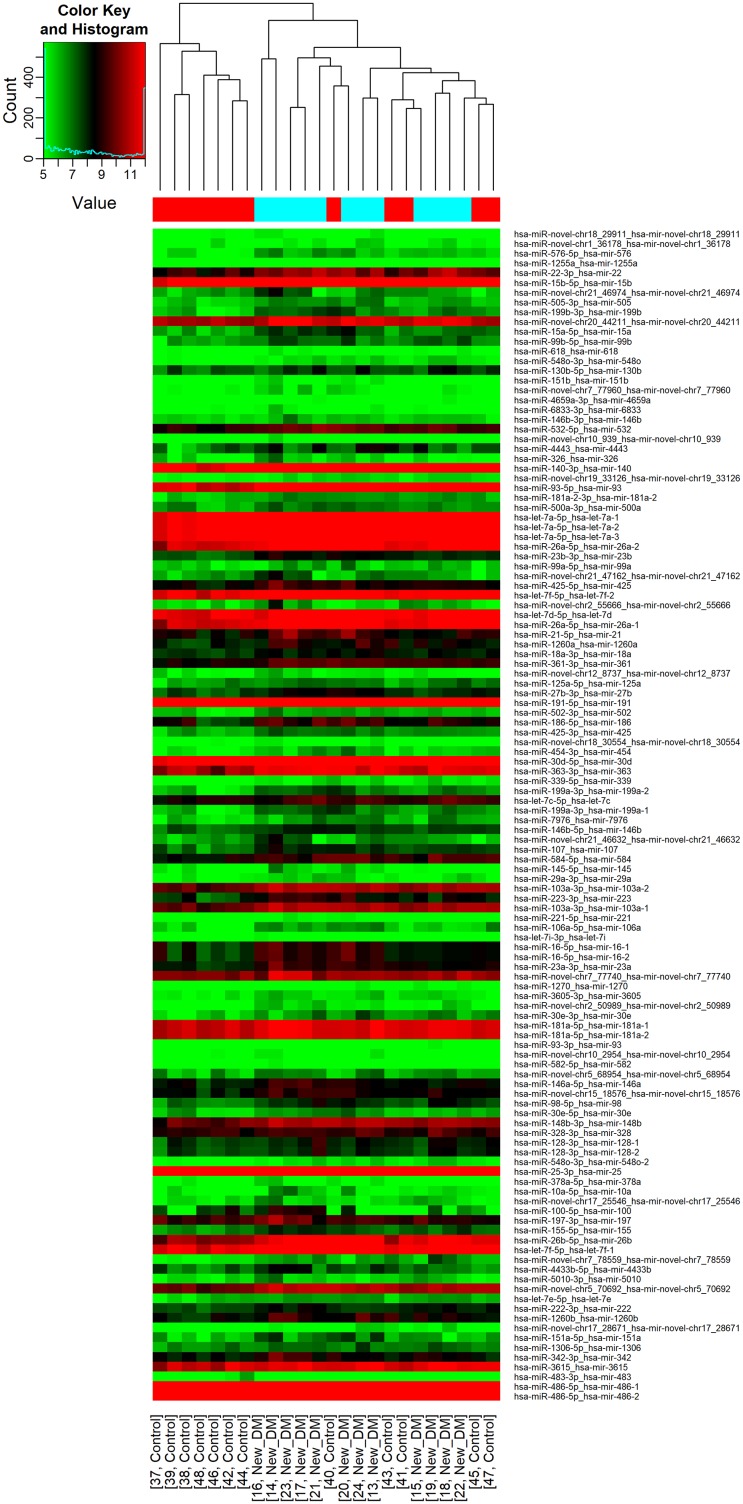

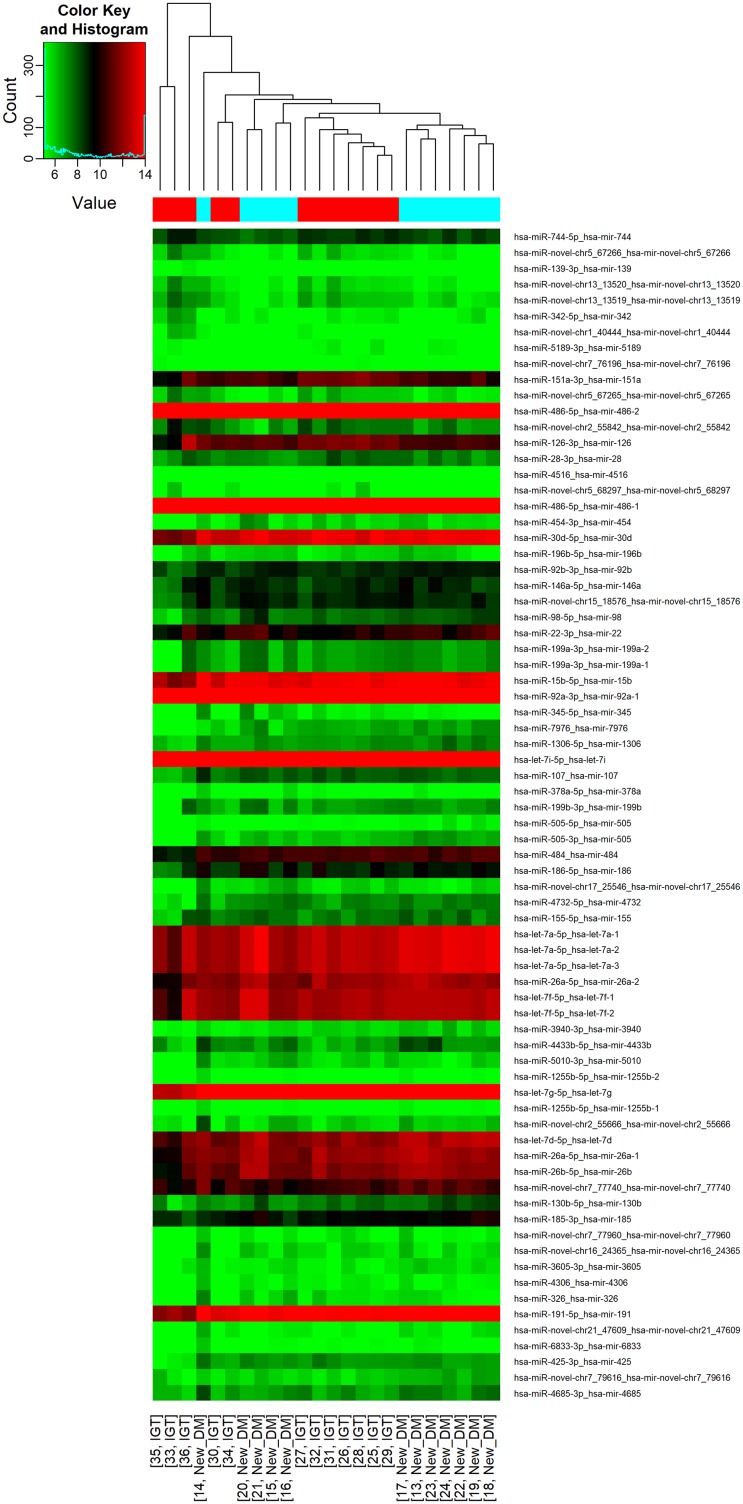
Differential miRNA expression in glucose tolerance groups The heatmap shows all differentially expressed miRNAs at adjusted p-value < 0.05. A. IGT versus NGT; B. screen-detected diabetes versus NGT; C. IGT versus screen-detected diabetes. Signal intensity was expressed as the log2 ratio between groups. Samples were grouped using hierarchical clustering based on similar expression profiles. Red, under-expression, white, no change, blue, overexpression.

**Figure 2:**
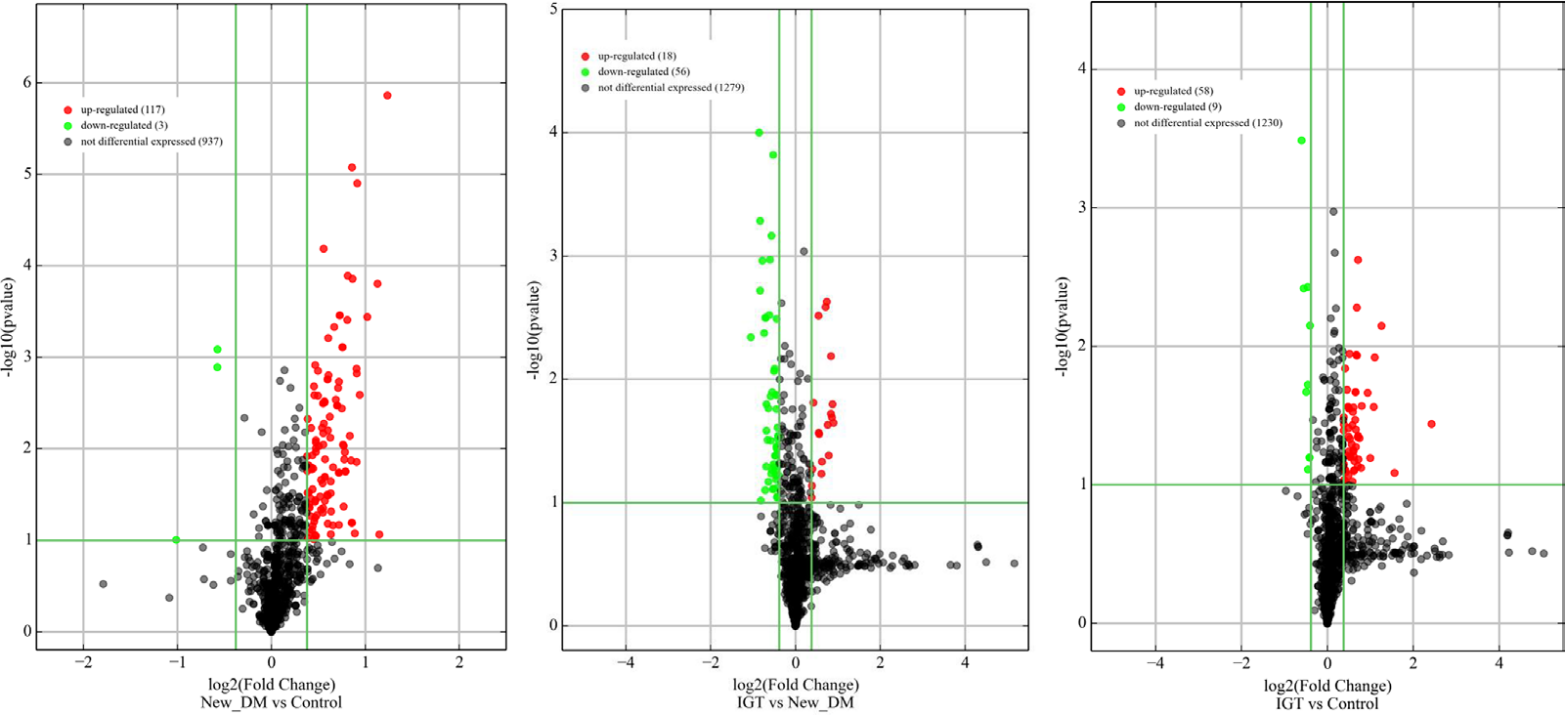
Volcano plots of precursor miRNA in individuals with screen-detected diabetes (DM), impaired glucose tolerance (IGT) compared to those with normal glucose tolerance (NGT) Differentially expressed miRNAs are those having fold changes ≥1.3, p-value ≤0.1.

All 36 samples (12 NGT, 12 IGT and 12 screen-detected diabetes) met the quality control standards. The clean reads (Adapter-trimmed reads) met and exceeded the specified sequencing coverage of 5 M (Supplementary Table 1). We generated Heat Map and Unsupervised Hierarchical Clustering on all expressed miRNAs (at least expressed in one sample) to produce miRNA or condition trees to allow us to pick out groups of similar miRNA. The result of hierarchical clustering on conditions showed a distinguishable miRNA expression profile among samples (Figure 1). For the identification of differentially expressed miRNAs, we computed “fold change” (i.e. the ratio of the group averages) and p-value between each group. miRNAs having fold changes ≥1.3, p-value ≤0.1 were selected as the differentially expressed miRNAs. Based on pre-specified criteria, we then used volcano plots to visualize the significantly differentially expressed pre-miRNAs between the study groups (Figure 2). In total, we identified a total of 261 significant pre-miRNAs that generated 237 mature miRNAs at varying levels of expression. Upregulated pre-miRNA (mature miRNA) were 117 (107) between screen-detected DM versus NGT, 58 (54) between IGT and NGT and 56 (50) between screen-detected DM and IGT, and downregulated miRNAs were respectively, 3 (2); 9 (7) and 18 (17). Fifty seven (22%) of the differentially expressed miRNAs were novel, of which 21 were upregulated in DM versus NGT, 19 in IGT versus NGT and 8 in DM versus IGT, whilst none were downregulated in DM versus NGT, 1 in IGT versus NGT and 8 in DM versus IGT. The details of the ten most differentially expressed miRNAs can be found in Supplementary Table 2 which shows that miR-15b-5p remained amongst the ten most differentially expressed miRNA when screen-detected diabetes was compared to either NGT or IGT. Among the down-regulated species, miR-486-5p was found in both screen-detected DM versus NGT and screen-detected DM versus IGT. A total of 28 pre-miRNA encoding 25 matured miRNAs were upregulated in both screen-detected DM and IGT when compared to NGT (Table 2). Among the ten most differentially expressed miRNAs in IGT versus NGT, eight (miR-99b-5p, -30e-3p, -1260a, -4443, -novel-chr2_50989, -1260b, -151a-5p, -miR-223-3) were also dysregulated in screen-detected DM when compared to NGT. Two of these, miR-novel-chr2_50989 and miR-223-3p were also found to be amongst the top ten differentially expressed miRNAs between screen-detected DM versus NGT.

**Table 2:** Dysregulated Pre miRNA and mature miRNA in screen-detected diabetes and impaired glucose tolerance versus normoglucotolerant individuals

Pre-miRNA accession number	Pre-miRNA	miRNA accession number	miRNA	DM vs NGT fold change	p-value	IGT vs NGT fold change	p-value
MI0000746	hsa-mir-99b	MIMAT0000689	hsa-miR-99b-5p	1,820588	0,000138758	2,959804	0,082063563
MI0000749	hsa-mir-30e	MIMAT0000693	hsa-miR-30e-3p	1,784615	0,007252301	2,398601	0,007101456
MYNO2438	hsa-mir-novel-chr2_50989	hsa-miR-novel-chr2_50989	hsa-miR-novel-chr2_50989	2,355212	1,37684E-06	2.0	0,064120232
MI0006394	hsa-mir-1260a	MIMAT0005911	hsa-miR-1260a	1,713994	0,01312321	2,151037	0,011997711
MI0016786	hsa-mir-4443	MIMAT0018961	hsa-miR-4443	1,551667	0,085964281	2,115556	0,027357273
MI0000300	hsa-mir-223	MIMAT0000280	hsa-miR-223-3p	1,920202	0,002582624	1,678788	0,046035966
MI0014197	hsa-mir-1260b	MIMAT0015041	hsa-miR-1260b	1,697833	0,009034611	1,917339	0,021651005
MI0000438	hsa-mir-15b	MIMAT0000417	hsa-miR-15b-5p	1,885503	1,26022E-05	1,409377	0,057613054
MYNO2570	hsa-mir-novel-chr20_44211	hsa-miR-novel-chr20_44211	hsa-miR-novel-chr20_44211	1,813224	8,42372E-06	1,60939	0,005237963
MYNO8	hsa-mir-novel-chr7_78559	hsa-miR-novel-chr7_78559	hsa-miR-novel-chr7_78559	1,801314	0,013459765	1,535304	0,062496157
MI0000809	hsa-mir-151a	MIMAT0004697	hsa-miR-151a-5p	1,705487	0,042803475	1,723404	0,075509343
MI0016746	hsa-mir-548o-2	MIMAT0005919	hsa-miR-548o-3p	1,657303	0,000348585	1,598315	0,011517573
MI0006402	hsa-mir-548o	MIMAT0005919	hsa-miR-548o-3p	1,657303	0,000348585	1,598315	0,011517573
MI0003772	hsa-mir-151b	MIMAT0010214	hsa-miR-151b	1,397004	0,053368366	1,58427	0,039862735
MYNO2414	hsa-mir-novel-chr1_36178	hsa-miR-novel-chr1_36178	hsa-miR-novel-chr1_36178	1,577617	0,015973544	1,483755	0,051048972
MI0001448	hsa-mir-425	MIMAT0003393	hsa-miR-425-5p	1,545474	0,007580947	1,366328	0,0612974
MYNO2060	hsa-mir-novel-chr5_70692	hsa-miR-novel-chr5_70692	hsa-miR-novel-chr5_70692	1,524669	0,001574976	1,405218	0,085501759
MI0000456	hsa-mir-140	MIMAT0004597	hsa-miR-140-3p	1,486817	0,013053757	1,521131	0,056333534
MI0000108	hsa-mir-103a-2	MIMAT0000101	hsa-miR-103a-3p	1,514849	0,001742993	1,445334	0,06271756
MI0000109	hsa-mir-103a-1	MIMAT0000101	hsa-miR-103a-3p	1,514822	0,001750868	1,445326	0,062651884
MYNO1801	hsa-mir-novel-chr17_28671	hsa-miR-novel-chr17_28671	hsa-miR-novel-chr17_28671	1,433333	0,033228045	1,51	0,029705075
MI0000289	hsa-mir-181a-1	MIMAT0000256	hsa-miR-181a-5p	1,465751	0,003192238	1,3978	0,02734155
MI0000269	hsa-mir-181a-2	MIMAT0000256	hsa-miR-181a-5p	1,465751	0,003192238	1,3978	0,02734155
MI0016005	hsa-mir-3615	MIMAT0017994	hsa-miR-3615	1,390032	0,009862178	1,42043	0,028236924
MYNO12	hsa-mir-novel-chr19_33126	hsa-miR-novel-chr19_33126	hsa-miR-novel-chr19_33126	1,410758	0,001410185	1,337408	0,094043894
MI0000269	hsa-mir-181a-2	MIMAT0004558	hsa-miR-181a-2-3p	1,34125	0,005944189	1,3025	0,077287593

### Quantitative real time PCR of MicroRNAs

**Figure 3:**
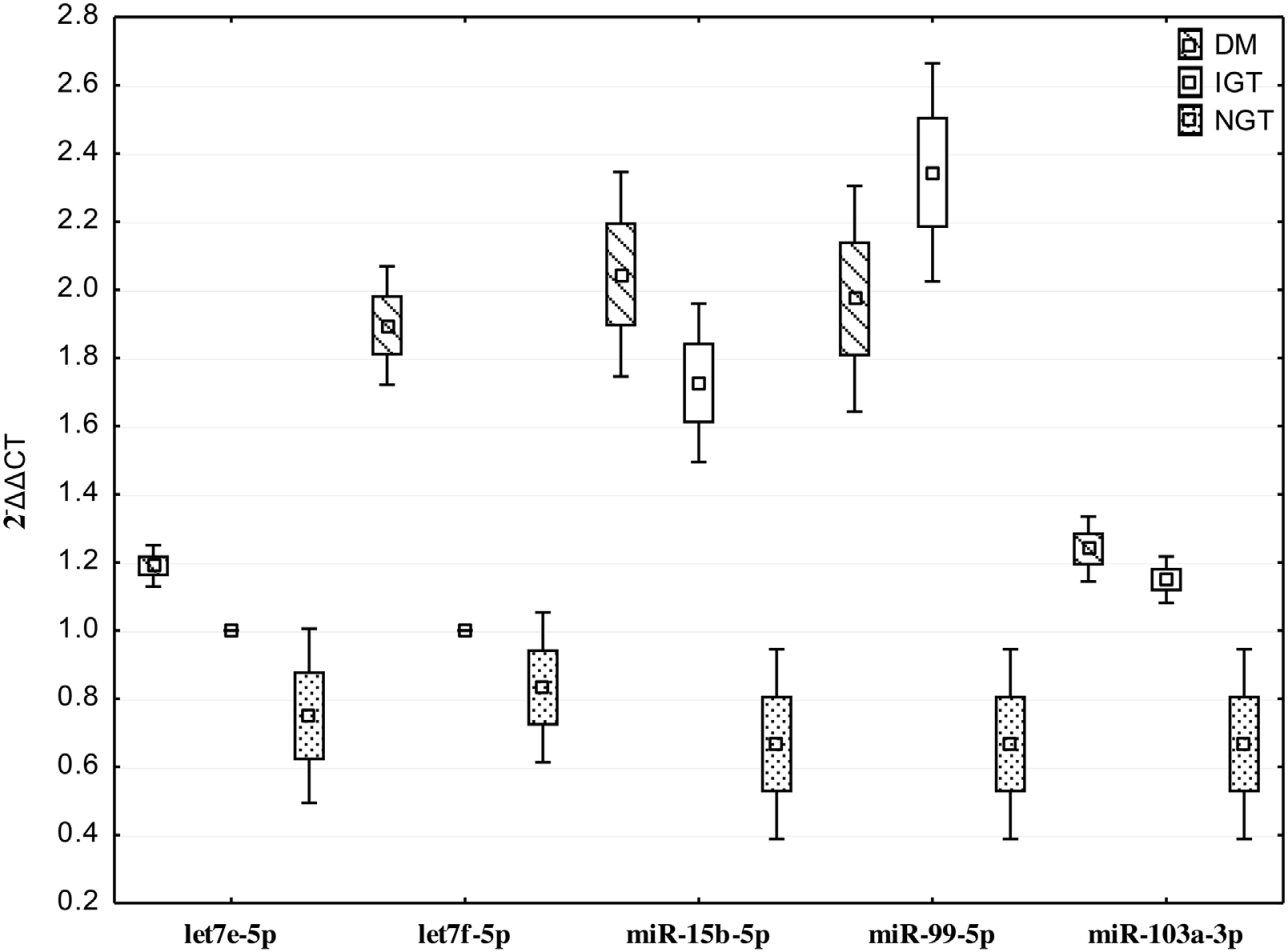
Quantitative real time PCR of differentially expressed miRNAs in individuals with screen-detected diabetes (DM), impaired glucose tolerance (IGT) and normol glucose tolerance (NGT) Data presented as mean and range.

As shown in Figure 3, we confirmed up-regulation of let7e-5p, let7f-5p, miR-15b-5p, miR-99b-5p, miR-103a-3p in subjects with diabetes. We also confirmed the non-expression of let7e-5p, let7f-5p in individuals with IGT.

### Target prediction and functional enrichment analysis

**Figure 4:**
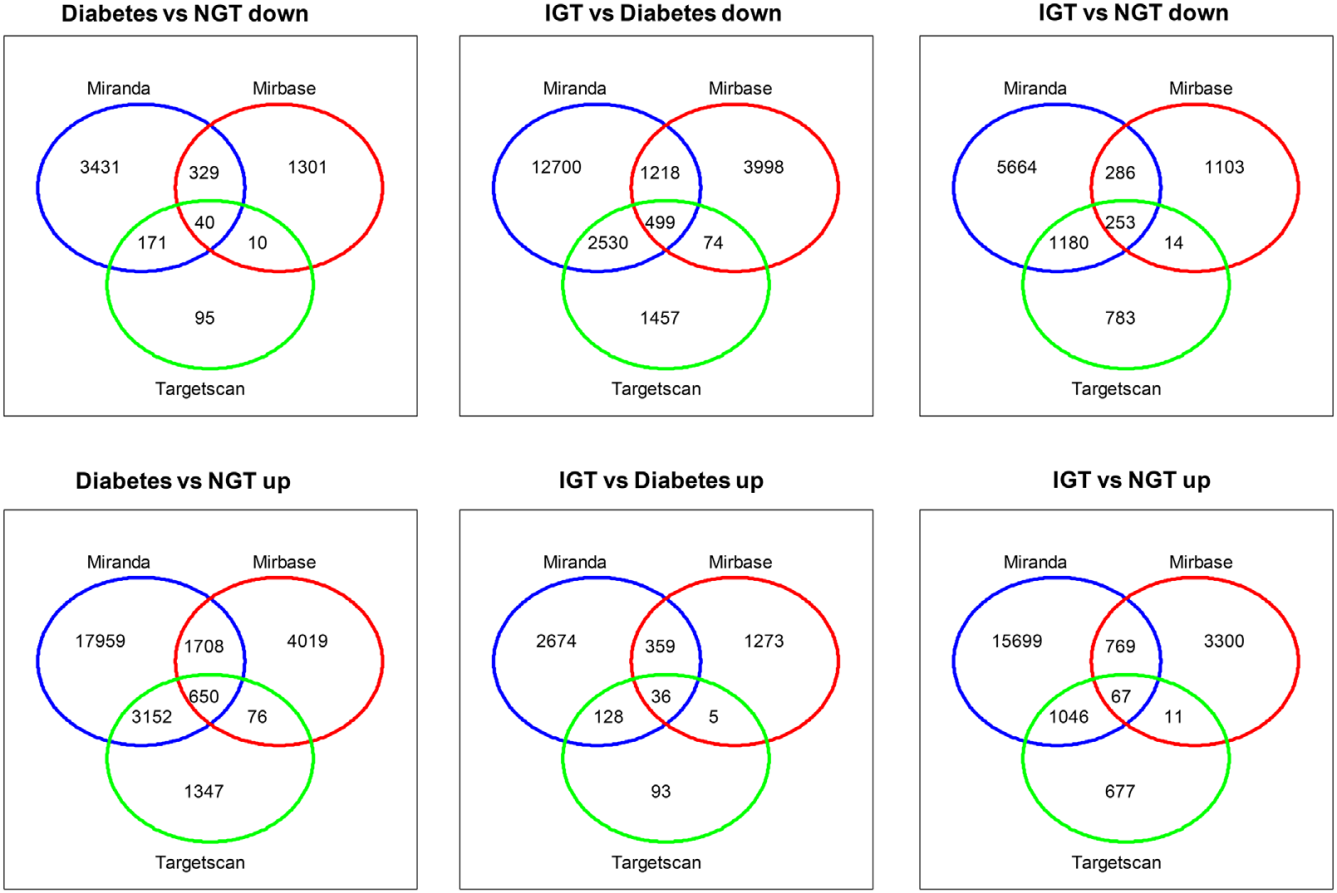
Venn diagrams showing target genes with three databases (TargetScan, Miranda and Microcosm v5) in individuals with screen-detected diabetes (DM), impaired glucose tolerance (IGT) compared to those with normal glucose tolerance (NGT)

We employed three databases namely, Mirbase, Miranda and Targetscan to conduct the analyses to improve the accuracy of our target site predictions. The target genes identified by the three databases were 690 in the screen-detected DM versus NGT, 535 screen-detected DM versus IGT, and 320 in IGT versus NGT. The predicted target sites of dysregluted miRNAs are shown in the Venn diagrams (Figure 4). The KEGG pathway analysis demonstrated 107 pathways (Supplementary Table 3). These pathways include those associated with signal transduction, cell-cell communications, cell growth and death, immune response, endocrine system and metabolic diseases.

## DISCUSSION

In this study, we employed high throughput sequencing to identify differentially expressed miRNAs associated with IGT and untreated diabetes in whole blood of South African mixed ancestry women, which in an earlier study we had established a high prevalence of undiagnosed IGT and DM [18). We observed evidence for differential expression of 61 in IGT, 109 in screen-detected diabetes both when compared to individuals with normal glucose tolerance, of which 25 were common in both conditions. Although several of these dysregulated miRNAs have been linked to diabetic and non-diabetic hyperglycaemia, we also uncovered 57 novel miRNAs. Of note is hsa-miR-novel-chr2_50989 which had the highest fold change in screen-detected DM and remained in the top ten differentially expressed miRNAs in IGT. Functional annotation of genes that are potentially regulated by the miRNAs implicated showed that signal transduction pathways (PI3K-Akt, MAPK, HIF-1, cAMP, FoxO, ErbB, Ras, Rap1 and insulin resistance); carbohydrate metabolism; glycan biosynthesis and metabolism, cell communication, cell growth and death; immune system; endocrine system and metabolic diseases are likely involved in the development of hyperglycaemia in this population.

Successful diabetes prevention implementation strategies rely on accurate identification of people with prediabetes, who are likely to progress to the full stage of time. To this end, the diagnosis of IGT for instance, requires individuals to be subjected to the cumbersome oral glucose overload and measurement of blood glucose post 2 hours [19, 20]. Consequently, individuals with this category of prediabetes largely remain undiagnosed. However, the emerging evidence for the role of miRNAs in the development of human diseases including diabetes is encouraging. In the development of diabetes, miRNAs have been reported to regulate metabolism, adipocyte differentiation, pancreatic development, β-cell mass, insulin biosynthesis, secretion, and signaling [21–25], underscoring their important role in the early diagnosis, pathogenesis and treatment of diabetes.

Some of the dysregulated miRNAs found in our study corroborate findings of many other studies that have aimed to characterize miRNAs in different tissue types of individuals with DM and/or prediabetes. A recent systematic study of dysregulated miRNAs in T2DM identified a total of 158 dysregulated miRNAs in adipose, islet, skeletal muscle, whole blood, PBMC, plasma and serum [26]. Similarly we found 36 (23%) of these miRNAs dysregulated in T2DM and IGT (Supplementary Table 4). Furthermore, three additional miRNAs (miR-27b, miR-98, and miR-21) previously reported to be dysregulated in mixed ethnic ancestry women with IGT or T2DM [27] were also differentially expressed in screen-detected DM in our sample. The miRNAs found in the current study and others have been shown to play a direct role in insulin production and secretion [21-25, 28]. This was confirmed by bioinformatics techniques we applied to identify the potential biological functions affected by the miRNA signatures. p53 signaling, PI3K/Akt, p53 signaling and MAPK were respectively the 2^nd^, 3^rd^ and 6^th^ targeted significant pathways in enrichment analysis by KEGG. The PI3K/Akt/ and MAPK pathways plays a major signaling role in the cellular response to extracellular stimuli, including glucose homeostasis, cell proliferation and survival [29]. In glucose homeostasis, the activation of these pathways is directly under the control of insulin receptors upon insulin stimulation [30]. A number of miRNAs such as the let-7 family, 30ep-5p [26, 31, 32] found in this study and others have been shown to be involved in these pathways. These miRNAs have be reported to exert their function by suppressing the expression of insulin receptor genes [17, 32].

Although many similarities were found between this study and others, our study is unique for uncovering that some of these miRNAs were differentially expressed between diabetic and non-diabetic dysglycaemia. Indeed, using OGTT to characterise asymptomatic participants, we identified three miRNAs that potentially distinguish between diabetic and non-diabetic hyperglycaemia. For example, miR-126-3p, and miR-28-3p were upregulated in IGT when compared to screen-detected DM, whilst miR-486-5p was down-regulated in screen-detected DM in comparison to either IGT or NGT. miR-126 is expressed by cells that modulate inflammatory response and vascular homeostasis through enhanced production of anti-inflammatory chemokines, and has been shown to be reduced in T2DM [33–37]. The downgrelation of miR-126 has been shown to be mostly pronounced in poorly controlled T2DM and in T2DM with complications when compared to sujects with T2DM without complication [38]. Similarly, in a study that investigated miR-126 in serum of DM patients with varying degrees of retinopathy, miR-126 was reduced in patients versus the controls, but lowest in patients with proliferative diabetic retinopathy [39]. Taken together, our findings of upregulated miR-126 and others in IGT versus screen-detected DM most probably point towards a cascading reduction with respect to diabetes related complications suggesting a potential role for miR-126 in distinguishing prediabetes from diabetes. Indeed, Liu et al [40]), examined the usefulness of miR-126 in predicting prediabetes and T2DM and reported lower levels in T2DM compared to prediabetes, even though both were significanlty lower than in healthy controls. It is important to note that a number of miRNAs including novel ones with potential to distinguish between hyperglycaemia and normal glucose tolerance were uncovered in the current study. For example, miR- hsa-miR-1299 had the highest fold change in IGT versus controls and was not detected in individuals with DM, whilst mir-novel-chr2_55842 was amongst the 10^th^ most differentially expressed in IGT only. In hepato-hepatocellular carcinoma, miR-1299 inhibits cell proliferation by targeting cyclin-dependent kinase 6, [41] however there is limited information about miR-1299 in diabetes. Therefore, further studies are needed to elucidate the molecular mechanisms of miR-1299 and other novel miRNAs identified in this study.

The major strengths of this study lies in the choice of high throughput sequencing which is a powerful strategy for identifying known and new candidate miRNAs related to the disease process. Furthermore, this method was ideal to cover unidentified miRNAs in this rather heterogeneous mixed ancestry population. Importantly, we did not include individuals on diabetes lowering treatment as this had the potential to bias our findings. Metformin, a drug that targets protein kinase activity and which is widely used for the treatment of insulin resistance, is believed to operate mainly by activating the insulin signaling pathway AMPK [29]. Lastly, our study participants were well-characterized by OGTT, and were matched for age, sex, blood pressure, serum cotinine and cholesterol. Therefore, we were able to uncover several known and novel miRNAs showing statistically significant evidence for differential expression by glucose tolerance status. In total we identified 57 novel differentially expressed miRNAs of which miR-novel-chr2_50989 deserves further consideration as this was amongst the top differentially expressed in both screen-detected and IGT. Despite these strengths, our study has limitations. Although our sample size is comparable to similar studies, we still consider it small. A small sample size has the potential to limit the ability to detect some significant differences in miRNA expression levels. Although, we employed RT-qPCR to confirm the expression of miRNAs identified by the deep sequencing approach, this was not performed in an independent sample. Therefore, a larger independent study is required to confirm our findings in this population. The cross-sectional design of this study limits our ability to draw conclusions with regards to association with disease progression, or to explore the role of particularly novel miRNAS in the molecular mechanisms of hyperglycaemia. Despite these considerations, several miRNAs whose molecular mechanism has been previously investigated were also found in this population group.

Overall, in addition to complementing earlier studies on miRNAs in prediabetes and diabetes, our findings provide evidence of known and novel differentially expressed miRNAs in African mixed ancestry individuals with IGT and screen-detected DM. We further observed that the aberrant expression profiles of miRNAs were linked to several biological processes, such as signal transduction, cell-cell communications, cell growth and death, immune response, endocrine system and metabolic diseases. Larger prospective studies in this and other racial populations from Africa are needed to characterize the molecular mechanisms of African-specific differentially expressed miRNAs, as well as assess their potential to predict worsening of glucose tolerance status.

## MATERIALS AND METHODS

### Ethical approval of the study

This investigation is based on the Cape Town Vascular and Metabolic Health (VMH) study, that has been approved by the Research Ethics Committees of the Cape Peninsula University of Technology (CPUT) and Stellenbosch University (respectively, NHREC: REC - 230 408 – 014 and N14/01/003). For this sub-study, ethical approval was also obtained from the CPUT Health and Wellness Sciences Research Ethics Committee (CPUT/HW-REC 2014/H08). The study was conducted according to the Code of Ethics of the World Medical Association (Declaration of Helsinki). All participants signed written informed consent after all the procedures had been fully explained in the language of their choice.

### Study design and procedures

This was a cross-sectional study involving participants from the ongoing Cape Town Vascular and Metabolic Health (VMH) study. VMH is an extension of the Cape Town Bellville South study, which has been described in details previously [42, 43]. Data collection was based on a standardised questionnaire available in the electronic form on a password-protected personal digital assistant (PDA). Upon completion of the assessment of each participant, data were automatically encrypted and transmitted via mobile internet connection to a dedicated server, from which they were checked for completion, downloaded and stored for future use. Physical examination involved data collection on blood pressure (BP) which was measured according to the World Health Organisation (WHO) guidelines [44], using a semi-automatic digital blood pressure monitor (Omron M6 comfort-preformed cuff BP Monitor) on the right arm in sitting position and at rest for at least 10 minutes. Body weight (to the nearest 0.1 kg) was measured with the subject in light clothing and without shoes, using an Omron body fat meter HBF-511digital bathroom scale, which was calibrated and standardized using a weight of known mass. Height to the nearest centimetre was measured with a stadiometer, with subjects standing on a flat surface. Body Mass Index (BMI) was calculated as weight per square meter (kg/m^2^). Waist circumference was measured with a non-elastic tape at the level of the narrowest part of the torso, as seen from the anterior view. BP and anthropometric measurements were performed three times and their average used for analysis.

All participants underwent a 75 g oral glucose tolerance test (OGTT) as recommended by the WHO [20]. Further, the following biochemical parameters were analyzed at an ISO 15189 accredited Pathology practice (PathCare, Reference Laboratory, Cape Town, South Africa): glycated haemoglobin (HbA1c) by High Perfomance Liquid Chromotography (Biorad Variant Turbo, BioRad, South Africa); serum insulin by a paramagnetic particle chemiluminescence assay (Beckman DXI, Beckman Coulter, South Africa); serum cotinine by Competitive Chemiluminescent (Immulite 2000, Siemens, South Africa); plasma glucose by enzymatic hexokinase method (Beckman AU, Beckman Coulter, South Africa); total cholesterol (TC); high density lipoprotein cholesterol (HDL-c) by enzymatic immunoinhibition – End Point (Beckman AU, Beckman Coulter, South Africa);; triglycerides (TG) by glycerol phosphate oxidase-peroxidase, End Point (Beckman AU, Beckman Coulter, South Africa); low density lipoprotein cholesterol (LDL) by enzymatic selective protection – End Point (Beckman AU, Beckman Coulter, South Africa); and ultrasensitive C-reactive protein (CRP) by Latex Particle immunoturbidimetry (Beckman AU, Beckman Coulter, South Africa). In addition, blood sample were collected in a Tempus RNA tube (Applied Biosystems) and stored at -80 degrees for RNA extraction and analysis. Participants selected for this analysis were 12 individuals with screen-detected diabetes, 12 with prediabetes and 12 with normal glucose tolerance, matched for age, blood pressure, smoking and body mass index.

### RNA isolation

Total RNA, including miRNA, was isolated from whole blood using MagMax total RNA isolation kit (ThermoFisher Scientific, South Africa) according to manufacture’s instructions. The concentration and purity of the RNA samples were determined using a NanoDrop ND-1000 spectrophotometer and RNA samples that met a 260/280 value >1.8 and a concentration > 20ug/ul were used for miRNA sequencing. Furthermore, RNA integrity was evaluated by denaturing agarose gel electrophoresis.

### miRNA sequencing

Small RNA library construction, deep sequencing, and data processing were performed at Arraystar Inc., Rockville, USA. Total RNA of each sample was used to prepare the miRNA sequencing library which included the following steps: 1) 3'-adapter ligation with T4 RNA ligase 2 (truncated); 2) 5'-adapter ligation with T4 RNA ligase; 3) cDNA synthesis with RT primer; 4) PCR amplification; 5) extraction and purification of ∼130-150 bp PCR amplified fragments (correspond to ∼15-35 nt small RNAs) from the PAGE gel. After the completed libraries were quantified with Agilent 2100 Bioanalyzer, the DNA fragments in the libraries were denatured with 0.1M NaOH to generate single-stranded DNA molecules, captured on Illumina flow cells, amplified *in situ* and finally sequenced for 51 cycles on Illumina HiSeq according to the manufacturer’s instruction. Raw sequences were generated as clean reads from Illumina HiSeq by real-time base calling and quality filtering. The clean reads that passed the quality filter were processed to remove the adaptor sequence as the trimmed reads. The trimmed reads (length ≥ 15 nt) were aligned to the human pre-miRNA in miRBase 21, using novoalign software. The miRNA expression levels were measured and normalized as transcripts per million of total aligned miRNA reads (TPM). miRNAs having fold changes >= 1.3, P-value <= 0.1 were selected as the differentially expressed miRNAs. Novel miRNAs were predicted by algorithms such as miRDeep [45].

### Analysis of individual miRNAs using quantitative reverse-transcription PCR (RT-qPCR)

To confirm the expression of miRNAs identified by the deep sequencing approach, RT-qPCR analysis was performed using miRNA from the same samples used in miRNA deep sequencing. miRNA was converted to cDNA using the TaqMan MicroRNA Reverse Transcription Kit according to the manufacturer's protocol (Life Technologies, USA). miRNA expression levels were assessed using and TaqMan miRNA Assay primers in conjuction with Quantum Studio 7 (Life Technologies, USA). The delta delta Ct (2^−ΔΔCT^) method was used to determine fold-change of miRNA expression between samples using the average expression of RNU6B, and miR-425 as endogenous controls. We selected the following miRNA for validation, let7e-5p, let7f-5p, miR-15b-5p, miR-99b-5p, miR-103a-3p.

### Target prediction and functional enrichment analysis

To improve the accuracy of messenger RNA (mRNA) gene targets, we used three different prediction algorithms, TargetScan (v6.2) (http://www.targetscan.org/vert_60/), Miranda, and Microcosm v5. The Venny tool (Venny v2.0.2) (http://bioinfogp.cnb.csic.es) was used to filter miRNA gene targets common to all three programs. miRNAs with the highest fold changes among the increased and decreased miRNAs were chosen for mRNA network analysis. These networks showed how one miRNA targets several mRNAs, which in turn can be targeted by several miRNAs and these are shown in Supplementary Figure 1. Commonly predicted gene targets were subjected to functional analysis using Kyoto Encyclopedia of Genes and Genomes (KEGG). We used a conservative Fisher’s exact test and the false discovery rate method to calculate the targeted pathways.

### Statistical analysis

Data were analysed using the R statistical software version 3.2.2 [2015-08-14], (The R Foundation for Statistical Computing, Vienna, Austria). Variables are summarized as mean and standard deviation or median [25^th^-75^th^ percentiles). The Shapiro-Wilk W test was employed to determine whether the data were normally distributed, based on probability thresholds of p>0.1.

## SUPPLEMENTARY MATERIALS FIGURE AND TABLES




