# Supplementary material for: MicroRNA profiling and their pathways in South African individuals with prediabetes and newly diagnosed type 2 diabetes mellitus
Source: Oncotarget. 2018 Jul 17;9(55):30485–98. doi: 10.18632/oncotarget.25271 (PMC6078144; doi:10.18632/oncotarget.25271)
Supplement: Supplementary file 1 [file oncotarget-09-30485-s001.pdf]

MicroRNA profiling and their pathways in South African individuals with prediabetes and newly diagnosed type 2 diabetes mellitus

SUPPLEMENTARY MATERIALS

Supplementary Table 1: The total numbers of the reads at the sequencing data processing

See Supplementary File 1

Supplementary Table 2: Top differential expressed miRNAs in diabetes and impaired glucose tolerance

See Supplementary File 1

Supplementary Table 3: Functional Enrichment analysis

See Supplementary File 1

Supplementary Table 4: Summary of miRNAs previously associated with T2DM

See Supplementary File 1

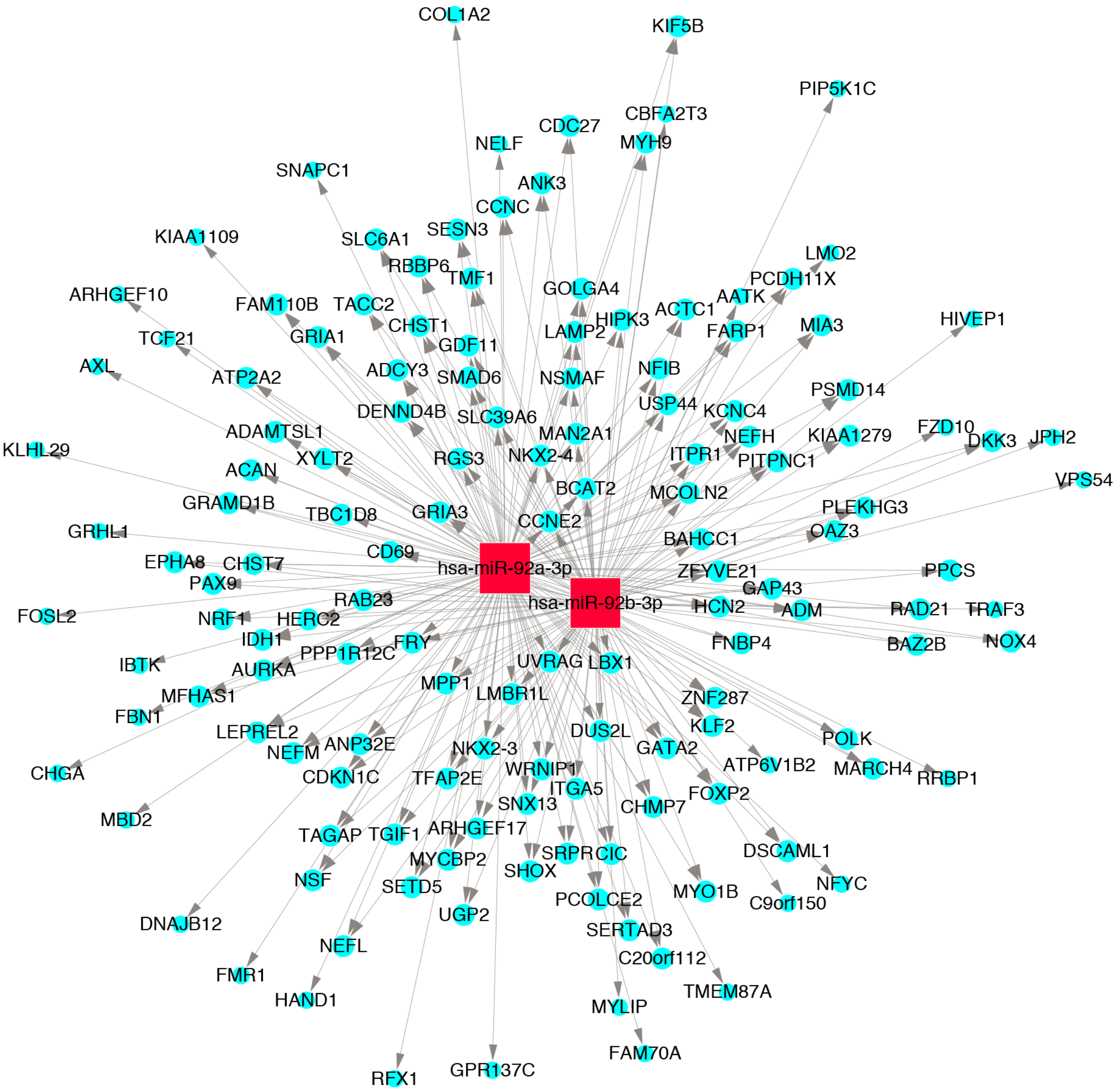

Supplementary Figure 1: Network of miRNA and mRNA. The following figure involves miR92a-3p and miR92b-3p and its target mRNAs. Blue circular nodes representing mRNAs, red rectangle node representing miRNAs).

REFERENCES

1. Pek SL, Sum CF, Lin MX, Cheng AK, Wong MT, Lim SC, Tavintharan S. Circulating and visceral adipose miR-100 is down-regulated in patients with obesity and Type 2 diabetes. Mol Cell Endocrinol. 2016; 427:112–23. https://doi.org/10.1016/j.mce.2016.03.010.
2. Karolina DS, Tavintharan S, Armugam A, Sepramaniam S, Pek SL, Wong MT, Lim SC, Sum CF, Jeyaseelan K. Circulating miRNA profiles in patients with metabolic syndrome. J Clin Endocrinol Metab. 2012; 97:E2271–76. https://doi.org/10.1210/jc.2012-1996.
3. Kameswaran V, Bramswig NC, McKenna LB, Penn M, Schug J, Hand NJ, Chen Y, Choi I, Vourekas A, Won KJ, Liu C, Vivek K, Naji A, et al. Epigenetic regulation of the DLK1-MEG3 microRNA cluster in human type 2 diabetic islets. Cell Metab. 2014; 19:135–45. https://doi.org/10.1016/j.cmet.2013.11.016.
4. Gallagher IJ, Scheele C, Keller P, Nielsen AR, Remenyi J, Fischer CP, Roder K, Babraj J, Wahlestedt C, Hutvagner G, Pedersen BK, Timmons JA. Integration of microRNA changes in vivo identifies novel molecular features of muscle insulin resistance in type 2 diabetes. Genome Med. 2010; 2:9. https://doi.org/10.1186/gm130.
5. Ortega FJ, Moreno-Navarrete JM, Pardo G, Sabater M, Hummel M, Ferrer A, Rodriguez-Hermosa JI, Ruiz B, Ricart W, Peral B, Fernández-Real JM. MiRNA expression profile of human subcutaneous adipose and during adipocyte differentiation. PLoS One. 2010; 5:e9022. https://doi.org/10.1371/journal.pone.0009022.
6. Locke JM, da Silva Xavier G, Dawe HR, Rutter GA, Harries LW. Increased expression of miR-187 in human islets from individuals with type 2 diabetes is associated with reduced glucose-stimulated insulin secretion. Diabetologia. 2014; 57:122–28. https://doi.org/10.1007/s00125-013-3089-4.
7. Belongie KJ, Ferrannini E, Johnson K, Andrade-Gordon P, Hansen MK, Petrie JR. Identification of novel biomarkers to monitor β-cell function and enable early detection of type 2 diabetes risk. PLoS One. 2017; 12:e0182932. https://doi.org/10.1371/journal.pone.0182932.
8. Santovito D, De Nardis V, Marcantonio P, Mandolini C, Paganelli C, Vitale E, Buttitta F, Bucci M, Mezzetti A, Consoli A, Cipollone F. Plasma exosome microRNA profiling unravels a new potential modulator of adiponectin pathway in diabetes: effect of glycemic control. J Clin Endocrinol Metab. 2014; 99:E1681–85. https://doi.org/10.1210/jc.2013-3843.
9. Klöting N, Berthold S, Kovacs P, Schön MR, Fasshauer M, Ruschke K, Stumvoll M, Blüher M. MicroRNA expression in human omental and subcutaneous adipose tissue. PLoS One. 2009; 4:e4699. https://doi.org/10.1371/journal.pone.0004699.
10. Nunez Lopez YO, Garufi G, Seyhan AA. Altered levels of circulating cytokines and microRNAs in lean and obese individuals with prediabetes and type 2 diabetes. Mol Biosyst. 2016; 13:106–21. https://doi.org/10.1039/C6MB00596A.
11. Kong L, Zhu J, Han W, Jiang X, Xu M, Zhao Y, Dong Q, Pang Z, Guan Q, Gao L, Zhao J, Zhao L. Significance of serum microRNAs in pre-diabetes and newly diagnosed type 2 diabetes: a clinical study. Acta Diabetol. 2011; 48:61–69. https://doi.org/10.1007/s00592-010-0226-0.
12. Seyhan AA, Nunez Lopez YO, Xie H, Yi F, Mathews C, Pasarica M, Pratley RE. Pancreas-enriched miRNAs are altered in the circulation of subjects with diabetes: a pilot cross-sectional study. Sci Rep. 2016; 6:31479. https://doi.org/10.1038/srep31479.
13. Liang GW, Song Y, Shao DH, et al. The change of serum miR-375 and miR-29a and their correlation with glycemic control and lipid profile in patients with newly diagnosed type 2 diabete. Chin. J. Lab. Diagn. 2013; 17:475–78.
14. Zampetaki A, Kiechl S, Drozdov I, Willeit P, Mayr U, Prokopi M, Mayr A, Weger S, Oberhollenzer F, Bonora E, Shah A, Willeit J, Mayr M. Plasma microRNA profiling reveals loss of endothelial miR-126 and other microRNAs in type 2 diabetes. Circ Res. 2010; 107:810–17. https://doi.org/10.1161/CIRCRESAHA.110.226357.
15. Meng S, Cao JT, Zhang B, Zhou Q, Shen CX, Wang CQ. Downregulation of microRNA-126 in endothelial progenitor cells from diabetes patients, impairs their functional properties, via target gene Spred-1. J Mol Cell Cardiol. 2012; 53:64–72. https://doi.org/10.1016/j.yjmcc.2012.04.003.
16. Jansen F, Wang H, Przybilla D, Franklin BS, Dolf A, Pfeifer P, Schmitz T, Flender A, Endl E, Nickenig G, Werner N. Vascular endothelial microparticles-incorporated microRNAs are altered in patients with diabetes mellitus. Cardiovasc Diabetol. 2016; 15:49. https://doi.org/10.1186/s12933-016-0367-8.
17. Yang Z, Chen H, Si H, Li X, Ding X, Sheng Q, Chen P, Zhang H. Serum miR-23a, a potential biomarker for diagnosis of pre-diabetes and type 2 diabetes. Acta Diabetol. 2014; 51:823–31. https://doi.org/10.1007/s00592-014-0617-8.
18. Long Y, Zhan Q, Yuan M, Duan X, Zhou J, Lu J, Li Z, Yu F, Zhou X, Yang Q, Xia J. The expression of microRNA-223 and FAM5C in cerebral infarction patients with diabetes mellitus. Cardiovasc Toxicol. 2017; 17:42–48. https://doi.org/10.1007/s12012-015-9354-7.
19. Li MY, Pan SR, Qiu AY. Roles of microRNA-221/222 in type 2 diabetic patients with post-menopausal breast cancer. Genet Mol Res. 2016; 15.
20. Olivieri F, Spazzafumo L, Bonafè M, Recchioni R, Prattichizzo F, Marcheselli F, Micolucci L, Mensà E, Giuliani A, Santini G, Gobbi M, Lazzarini R, Boemi M, et al. MiR-21-5p and miR-126a-3p levels in plasma and circulating angiogenic cells: relationship with type 2

diabetes complications. Oncotarget. 2015; 6:35372–82. https://doi.org/10.18632/oncotarget.6164.

21. Yan ST, Li CL, Tian H, Li J, Pei Y, Liu Y, Gong YP, Fang FS, Sun BR. MiR-199a is overexpressed in plasma of type 2 diabetes patients which contributes to type 2 diabetes by targeting GLUT4. Mol Cell Biochem. 2014; 397:45–51. https://doi.org/10.1007/s11010-014-2170-8.
22. Zhou B, Li C, Qi W, Zhang Y, Zhang F, Wu JX, Hu YN, Wu DM, Liu Y, Yan TT, Jing Q, Liu MF, Zhai QW. Downregulation of miR-181a upregulates sirtuin-1 (SIRT1) and improves hepatic insulin sensitivity. Diabetologia. 2012; 55:2032–43. https://doi.org/10.1007/s00125-012-2539-8.
23. Wang C, Wan S, Yang T, Niu D, Zhang A, Yang C, Cai J, Wu J, Song J, Zhang CY, Zhang C, Wang J. Increased serum microRNAs are closely associated with the presence of microvascular complications in type 2 diabetes mellitus. Sci Rep. 2016; 6:20032. https://doi.org/10.1038/srep20032.
24. Corral-Fernández NE, Salgado-Bustamante M, Martínez-Leija ME, Cortez-Espinosa N, García-Hernández MH, Reynaga-Hernández E, Quezada-Calvillo R, Portales-Pérez DP. Dysregulated miR-155 expression in peripheral blood mononuclear cells from patients with type 2 diabetes. Exp Clin Endocrinol Diabetes. 2013; 121:347–53. https://doi.org/10.1055/s-0033-1341516.
25. Pan J, Chen H. Expression of miR-155 and miR-146a in patients with type 2 diabetes mellitus and its significance. Zhejiang Clin. Med. 2014; 16:1042–43.
26. Rong Y, Bao W, Shan Z, Liu J, Yu X, Xia S, Gao H, Wang X, Yao P, Hu FB, Liu L. Increased microRNA-146a levels in plasma of patients with newly diagnosed type 2 diabetes mellitus. PLoS One. 2013; 8:e73272. https://doi.org/10.1371/journal.pone.0073272.
27. Prabu P, Rome S, Sathishkumar C, Aravind S, Mahalingam B, Shanthirani CS, Gastebois C, Villard A, Mohan V, Balasubramanyam M. Circulating MiRNAs of 'Asian Indian Phenotype' Identified in Subjects with Impaired Glucose Tolerance and Patients with Type 2 Diabetes. PLoS One. 2015; 10:e0128372. https://doi.org/10.1371/journal.pone.0128372.
28. Liu Y, Gao G, Yang C, Zhou K, Shen B, Liang H, Jiang X. The role of circulating microRNA-126 (miR-126): a novel biomarker for screening prediabetes and newly diagnosed type 2 diabetes mellitus. Int J Mol Sci. 2014;15:10567-77. https://doi: 10.3390/ijms150610567.
29. Lu SY, Lu ZH, Tan L, Wan S. Association of Plasma miR-375 and miR-126 in patients with type 2 diabetes mellitus. Xiandai Jianyan Yixue Zazhi. 2014; 29:18–21.
30. Rezk NA, Sabbah NA, Saad MS. Role of MicroRNA 126 in screening, diagnosis, and prognosis of diabetic patients in Egypt. IUBMB Life. 2016; 68:452–58. https://doi.org/10.1002/iub.1502.
31. Ren YH, Shi XX, Yan DD, Li XL. The change of expression level of circulating miRNA-126 in patients with type 2 diabetes and its relative factors. Clin. J. Diabetes. 2014; 22:633–36.
